# Interplay of constipation, intestinal barrier dysfunction and fungal exposome in aetiopathogenesis of Parkinson’s disease: hypothesis with supportive data

**DOI:** 10.1042/BCJ20240621

**Published:** 2025-06-11

**Authors:** Chianna Umamahesan, Aleksandra Pilcicka, Jennifer Yick, Kieran Baker, Melvyn Smith, David Taylor, Yun Ma, Benjamin H. Mullish, Julian R. Marchesi, Steven Gilbert, Shervin D. Sadeghi Nasab, David Moyes, Polychronis Pavlidis, Bu’Hussain Hayee, Sylvia M. Dobbs, R. John Dobbs, André Charlett

**Affiliations:** 1 Host Microbiome Interaction: Clinical Pharmacology and Therapeutics, Institute of Pharmaceutical Science, King’s College London, London, U.K.; 2 The Maudsley Hospital, London, U.K.; 3 Centre for Host-Microbiome Interactions, King’s College London, London, U.K.; 4 Microbiology, King’s College Hospital, London, U.K.; 5 Institute of Liver Studies, King's College Hospital, London, U.K.; 6 Division of Digestive Diseases, Department of Metabolism, Digestion and Reproduction, Imperial College, London, U.K.; 7 Gastroenterology, King’s College Hospital, London, U.K.; 8 Statistics, Modelling and Economics, UK Health Security Agency, London, U.K.

**Keywords:** aetiopathogenesis, constipation, fungal load, intestinal barrier dysfunction, mycobiome, Parkinson’s disease

## Abstract

Constipation is a forerunner to Parkinson’s disease (PD) diagnosis, worsening thereafter. We explore the relationship of intestinal barrier dysfunction to constipation and whether intestinal fungal load is an aggravating factor. Fungal load was quantified by real-time PCR, using ITS1F-ITS2 primer set, on microbial DNA extract from stool in 68 participants with PD, 102 without. Fungal load was 60% higher per decade after age 60 years, with no PD status interaction with age. After age adjustment, it was associated inversely with dietary renal acid load. It was unrelated to the presence of constipation or barrier dysfunction. Neither consumption of antimicrobials nor of other targeted exogenous substances was associated. Enzyme-linked immunosorbent assays measured barrier dysfunction markers, faecal alpha-1 antitrypsin (AAT), zonulin and serum intestinal fatty acid-binding protein (I-FABP). Barrier dysfunction was associated with constipation and slower radiographic colonic transit. Functional constipation was 28% more frequent with a doubling of AAT concentration. More colonic-transit test markers were retained in the transverse colon, the higher the AAT and zonulin concentrations, anatomically spotlighting abnormality for the entire colon. In contrast, the concentration of the small intestinal barrier marker I-FABP was associated with looser stool consistency, which is consistent with secondary microbial overgrowth. By showing a relationship of intestinal barrier dysfunction to constipation, this study supports the hypothesis that dysfunction may be consequential. Dysfunction may be a necessary, but not sufficient, precursor to PD, in allowing inflammaging. Since ageing is the clearest risk for PD, a gut pathogen escalating in abundance from the sixth decade, integral to fungal load, and whose reproduction and virulence is favoured by alkalinity, tallies.

## Introduction

We explore whether barrier dysfunction is a consequence of slow intestinal transit. Constipation is prodromal by decades to the clinical diagnosis of Parkinson’s disease (PD) [1]. The odds of having delayed colonic transit are doubled in those with PD compared with those without, the transverse colon being particularly affected [2]. Indeed, in PD, segmental delay in the transverse colon appeared susceptible to an ageing effect. An interrupted time series showed that, in PD, objectively measured flexor rigidity increased by 6% per year before the introduction of (non-stimulant) maintenance laxative and plateaued thereafter [3]. Thus, the gut microbiome of constipation appears to have a continuing and profound adverse effect, which is, at least in part, reversible. Irrespective of PD, potentially beneficial short chain fatty acid (SCFA) producing bacteria were significantly reduced in constipated patients, and pathogenic bacteria and fungi increased, particularly where constipation was severe [4]. Laxative treatment resulted in ‘normalisation’ of the faecal microbiota. Here, we confine ourselves to what is driving the process, rather than the spread as described by the pattern of neuronal loss in PD and staged distribution in subtypes [5-8].

PD has been described as a condition of premature or accelerated ageing. It fits with ‘inflammaging’ [9]. The age-related increase in serum concentration of the pro-inflammatory cytokine, interleukin-6 (IL-6), is premature by 10 years in PD [10]. Moreover, IL-6 concentration, in serum obtained four years earlier, predicts incident PD [11]. Intestinal inflammation, in a setting of compromised barrier function and increased cross-barrier translocation [12], may drive that systemic inflammation and consequent neuroinflammation and neuronal death. Indeed, a marker of migration of neutrophils into intestinal mucosa, faecal calprotectin, was 44% greater in PD, over and above an ageing effect and after adjustment for medicinal provocateurs (proton pump inhibitors [PPIs], anti-microbials) [13]. Indeed, we have suggested that the Parkinsonian syndrome is a *forme fruste* of systemic inflammatory response syndrome. Pulse rate was faster in PD, irrespective of postural fall in blood pressure, and despite any cardiac autonomic neuropathy [13]. Rate was related inversely to faecal concentrations of anti-fungal molecules (an imidazole-ring compound and benzoic acid); an anti-inflammatory (nicotinic acid, a form of vitamin B3); a barrier function protector (hypoxanthine); and an osmolyte which maintains cell integrity and influences protein folding (homarine). In explaining the faster pulse, a higher serum chemokine (C-C motif) ligand 20 (CCL20) concentration, indicative of chemotaxis for lymphocytes/dendritic cells towards epithelium, complemented the faecal imidazole deficit. A higher CCL20 was also linked to prolonged colonic transit, as were the deficits in faecal SCFAs.

A preliminary systematic review (Supplementary File S1) shows that, in PD, the alimentary fungal community (mycobiome) has been neglected compared with the bacteriome. We address whether fungal infection is more common in PD, and what differences in mycobiota does PD status confer. Of interest is that fungal colonisation of the gastrointestinal tract [14] is reported to reflect clinical disease activity in colitis [13] and flag disease in immunocompromised individuals [15]. Fewer than 1% of the microbes in the human intestinal tract are fungi, but their cells are more than 100 times larger than typical bacterial cells [16,17]. They provide surface area for host–microbe and inter-microbial kingdom interactions, as well as unique metabolic functions. Knowledge of fungi capable of growing in and colonising the gut is limited to a small number of species [18,19]. Their abundance may be influenced by oral hygiene and diet. The effect of demographic differences, even in age, on fungal load is ill-defined. The gut mycobiome appears less stable than the bacteriome.

**Figure 1:**
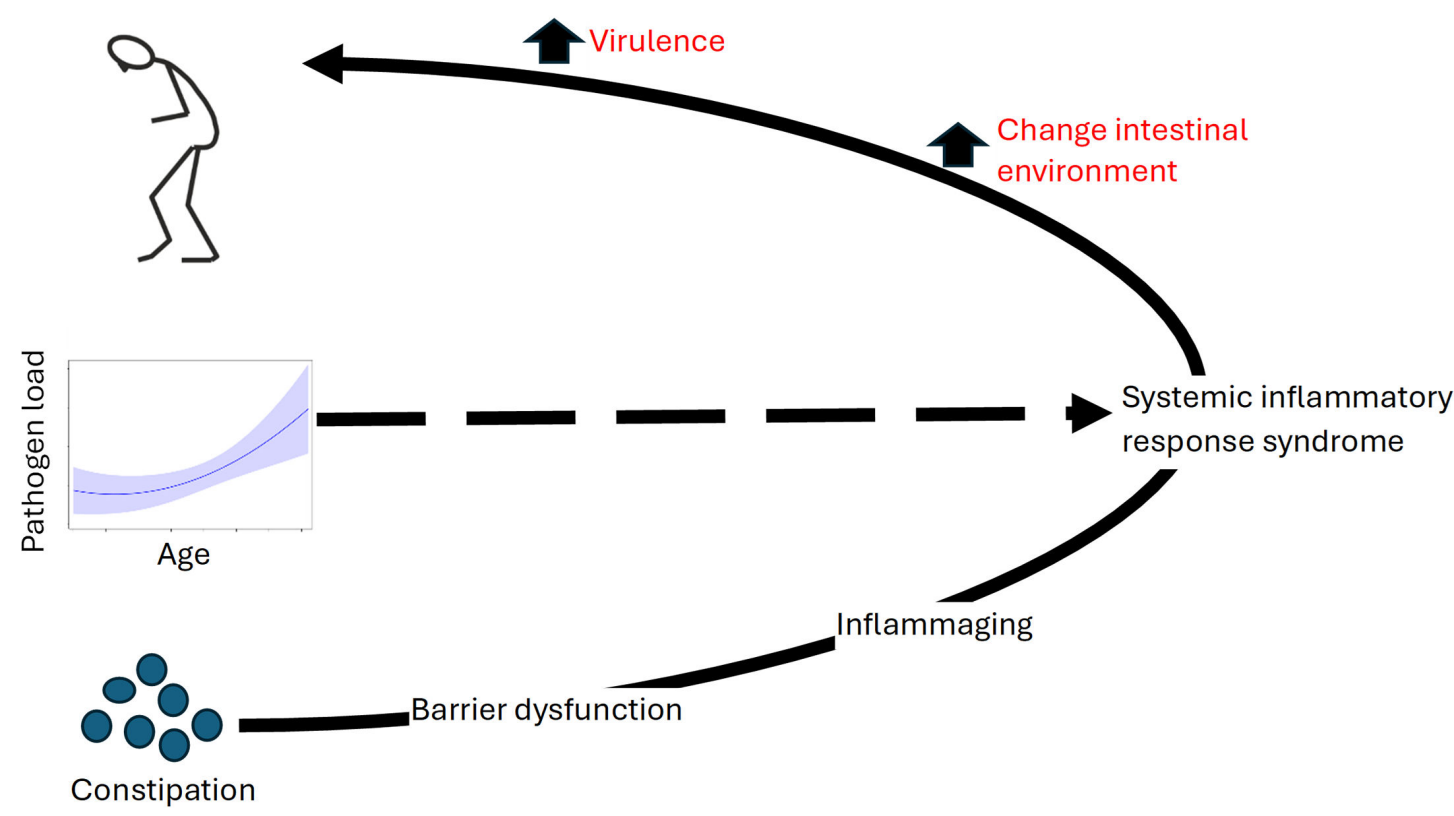
Hypothesis for aetiopathogenesis of Parkinson’s disease. Chronic constipation causes intestinal barrier dysfunction, with consequent inflammaging. Age-related change in load of faecal pathogens drive a systemic inflammatory response syndrome in this setting. Subsequent changes in gut environment may be selective for virulence.

We postulate (**Figure 1**) that chronic constipation may cause intestinal barrier dysfunction, which results in gut pathogen-driven inflammaging. A pathogen, which increases in load with age, may drive a systemic inflammatory response syndrome underlying PD, age being the biggest risk factor for PD. We question whether faecal fungal load may be that aggravating factor transforming a ‘leaky gut’ into PD. Subsequent alteration in the gut environment could be selective for microbial virulence.

## Materials and methods

### Cohort details

The observational study was set in a National Gut-Brain Axis research clinic. People with diagnosed PD were invited to volunteer. Inclusion was according to UK Brain Bank Criteria with at least three supportive criteria [20], after exclusion of causes of secondary Parkinsonism. Evidence of responsiveness to levodopa was not required. Probands’ cohabiting life partners were also invited to enlist. ‘Controls-proper’ neither had diagnosed PD nor resided with anyone who did. Website recruitment was complemented by proband dissemination, the latter also facilitating control recruitment. The study was approved by King’s College London Research Ethics Committee, with participants giving written informed consent.

All participants underwent subjective and objective assessments of clinical phenotype on recruitment. Number of antimicrobial courses in the last three years, and time since last course were recorded, as were current consumption of PPIs and NSAIDs, and any history of tobacco smoking. The Oral Health Impact Profile (OHIP-14) [21] was used to quantify the extent of oral disorders in terms of dysfunction, discomfort and disability. Faecal samples were collected by participants prior to arrival, or on-site, using a standardised kit (Fe-Col, Faecal collection device: Alpha Laboratories Ltd, Eastleigh, England; Faeces tube 101 × 16.5 mm, with short special spoon to collect a defined faeces sample: Sarstedt AG & Co, Nümbrecht, Germany). Kits were previously posted with instructions stressing that delivery must be within 4 h of evacuation (time noted). Participants unable to comply with instructions that day did so in the subsequent few days. Blood was collected in clot activator/serum separating tubes and allowed to stand for 15–30 minutes before centrifuging (4°C for 15 min) at 2500×g. Faecal and serum (250 µl aliquots) samples were frozen immediately at –80°C. Repeat assessment days were a median of one year apart.

### Immunoassays for barrier dysfunction markers

Faecal α-1-antitrypsin (AAT) and zonulin (Immundiagnostik AG, Bensheim, Germany) and intestinal fatty acid-binding protein (I-FABP) in serum (Abcam plc, Cambridge, UK) were used as markers of barrier dysfunction. Samples were assayed in duplicate. Optimal standard-curve fits for these enzyme-linked immunosorbent assays (ELISAs) were log_e_ concentration against optical density for zonulin and AAT assays; a non-transformed linear fit for I-FABP. Intraclass correlations (95% CI) for repeats were excellent: 0.98 (0.97, 1.00); 0.96 (0.92, 1.00); 0.97 (0.94, 1.00), for faecal zonulin, AAT and serum I-FABP antibody, respectively.

### Fungal load measurement

*Candida albicans* (source [22]) was grown on yeast extract (10%), peptone (20%), dextrose (20%) (YPD) medium, with Agar (BactoAgar; Difco) 20 g/l added as required. The *C. albicans* species cultured was inoculated on a YPD agar plate, incubated for one day at 37°C and, once fully grown, transferred to a 4°C refrigerator to halt growth. The day before DNA extraction, one colony from each plate was placed in 10 ml of YPD liquid medium in a 50 ml microcentrifuge tube. Tubes were incubated overnight in a shaking incubator at 30°C, then centrifuged at 3220×g for 1 minute and the supernatant discarded. Fungal cells were washed twice with phosphate-buffered saline.

Fungal DNA was extracted using the DNeasy PowerSoil Pro (Qiagen, Germany) kit. The bead-beating step was modified, taking further account of fungal cell wall thickness: 0.5 mm Yttria-stabilised zirconium oxide beads (Lysing Matrix Y, MP Bio) were substituted, to improve cell wall cracking and minimise shearing. Fungal cells were homogenised for a total of 3 minutes comprised of 20 second periods, with 5 minutes rest periods on ice between to minimise DNA heat damage. Extracted DNA concentrations and estimated purity of nucleic acids were calculated using a ND*-*1000 spectrophotometer (NanoDrop, Thermo Scientific). Ratios of *A*_260_/*A*_280_ > 1.8 and *A*_260_/*A*_230_ > 2.0 were accepted. All DNA extracts were stored at −20°C.

The forward primer used was ITS1F: CTT GGT CAT TTA GAG GAA GTA A, the reverse primer ITS2: GCT GCG TTC TTC ATC GAT GC (Eurofins Genomics, Germany). A 10-point standard curve of 10-fold serial dilutions of neat *C. albicans* DNA was run to determine the cut-point where genomic amplification by the primer set ceases to predict fungal DNA concentration reliably, taking into account triplicate reliability and reaction efficiency. Fungal DNA could not reliably be amplified at the lowest concentration (0.1 fg/μl). Removal of this point in the standard curve gave an overall qPCR reaction efficiency of 0.98, R^2^ of 0.99.

Serial dilutions of stock *C. albicans* (stored at −20°C) were prepared to give a standard 10-fold dilution series corresponding to 10^−8^, 10^−7^, 10^−6^, 10^−5^, 10^−4^, 10^−3^, 10^−2^, 10^−1^ and 1 genome copies/reaction of *C. albicans* DNA and used as standards (three replicates of each dilution) in each qPCR run. Known DNA concentrations were plotted against their threshold Ct values to obtain the standard curve.

The quantitative PCR protocol was executed on the Rotor-gene RG-6000 (Corbett Research). Each 10 μl reaction volume contained 6.2 μl RNA-free water; 2 μl HOT FIREPol EvaGreen qPCR Supermix (fluorescent dye); 0.4 μl ITS1F forward primer (Thermo Scientific) 1:10 dilution; 0.4 μl ITS2 reverse primer (Thermo Scientific) 1:10 dilution; and 1 µl of DNA template. Cycle parameters were an initial incubation step at 95°C for 12 minutes; followed by 45 cycles of denaturation at 95°C for 10 seconds; annealing at 57°C for 15 seconds; an extension at 72°C for 20 seconds; then a melting ramp from 57°C to 95°C.

After analysis of each batch of participant samples, the following data were extracted: (i) participant codes; (ii) known concentrations of serial dilutions used for *C. albicans* standard curve; (iii) Ct values for both standard curve and participants’ samples; (iv) qPCR efficiency coefficient; (v) predicted concentration of fungal DNA for each participant sample. Using the standard curve, the Ct value for each participant aliquot was interpreted as the concentration of fungal genomic DNA, expressed in terms of *C. albicans*.

Using the natural log of concentration, reliability of in-triplicate replicates was excellent (intraclass correlation 0.979 (0.972, 0.985), reliability 0.992), while reliability of repeats was moderate (intraclass correlation 0.517 (0.345, 0.690), reliability 0.684).

### Statistical analysis

The distribution of faecal fungal load was described by disease status, demographic characteristics, measurements of constipation and intestinal barrier dysfunction. All measurement occasions were used in linear mixed effects models, with a random intercept for subject included, to account for dependencies between longitudinal measurements on the same individuals. Demographic characteristics and exogenous substance consumption/smoking were considered as potential time-varying or fixed confounders. In addition, previously reported [13] nutritional intake, colonic transit and faecal metabolite measurement data in this cohort have been integrated (see Supplementary File S1). Disease status interaction terms were included in regression models, recognising that non-significance might result from insufficient power. A similar approach was taken to describing barrier dysfunction. Variables exhibiting a positively skewed distribution were converted to approximate normality by natural logarithmic transformation. Models were fitted within Stata 17 (StataCorp, College Station, Texas).

The study was conceived to generate hypotheses rather than as a pivotal study which tests pre-defined research hypotheses. The cohort size was, thus, determined on available resources and feasibility rather than on statistical sample size considerations.

## Results

### The cohort

**Table 1** gives the characteristics of 170 participants (68 with diagnosed PD and 102 without [referred to as the ‘remainder’]), in whom a faecal fungal load measurement for their first visit was available. There was no significant difference in age, height, weight or body mass index between those with and without PD but proportionally more males in the PD group (*P*<0.05). There were no participants receiving immunosuppressive therapy or medicinal treatment for diabetes.

**Table 1:** Participant characteristics.

Characteristics	Median (lower, upper quartile)^1^ at first assessment	
	PD (*n* = 68)	Remainder (*n* = 102)
**Demographic**		
Age (years)	69.5 (62, 74)	66 (60, 70)
Sex (male)	60^1^	43^1^
Height (cm)	170 (161, 177)	170 (162, 178)
Weight (kg)	71 (63, 84)	72 (63, 82)
Body mass index (kg/m^2^)	24.9 (22.8, 27.0)	24.4 (22.4, 26.7)
**Exogenous substance consumption**		
Anti-Parkinsonian medication (yes)	76^1^	0^1^
Laxatives (yes)	57^1^	14^1^ ^2^
Proton pump inhibitor (yes)	19^1^	11^1^
Non-steroidal anti-inflammatory drug (yes)^2^	7^1^	7^1^
Number of antimicrobial courses in last 3 years	1.0 (0.0, 2.0)	0.0 (0.0, 1.0)
Time since last antimicrobial course (years)	0.5 (0.0, 3.0)	1.0 (0.1, 3.0)
Total life-time experience of tobacco smoking (years)	1 (0, 15)	0 (0, 10)
Time since tobacco smoking abandoned (years)^3^	30 (22, 42)	28 (21, 40)
**PD descriptors**		
Time since diagnosis (years)	5 (2, 11)	-
Hoehn and Yahr functional rating	2 (2, 2)	-
UPDRS^4^ motor score severity rating	30 (20, 45)	-
**Bowel function**		
Colonic transit^5^: total number of markers-retained	23 (15, 36)	11 (7, 16)
Number markers-retained in transverse colon	4 (0, 9)	2 (1, 4)
Rome III: functional constipation (yes) [22]	42^1^	19^1^
Bristol stool scale (1–7 for specimen day) [23]	4 (2, 4)	3 (2, 4)
**Nutrient intake (5 day diary)** ^5^		
Energy intake (kcal/day)	7514 (6407, 9601)	7683 (6458, 8754)
Carbohydrate (g/day)	201 (176, 242)	179 (144, 223)
Free sugars (g/day)	32 (22, 52)	24 (19, 37)
Fat (g/day)	73 (61, 101)	73 (59, 87)
Cholesterol (mg/day)	230 (172, 319)	237 (167, 278)
Protein (g/day)	71 (60, 87)	76 (61, 95)
Fibre (g/day)	21 (17, 26)	20 (15, 25)
Alcohol (g/day)	4 (0, 11)	13 (3, 23)
Caffeine (mg/day)	116 (79, 199)	182 (135, 243)
Water (ml/day)	2058 (1778, 2434)	2328 (1891, 2814)
Potential Renal Acid Load (PRAL)	8 (-2, 97)	9 (-5, 75)
**Oral health impact profile**		
OHIP-14	0 (0, 4)	0 (0, 2)
**Barrier dysfunction markers**		
Serum intestinal fatty acid-binding protein 2 (pg/ml)^6^	454 (345, 588)	436 (330, 668)
Zonulin (ng/ml)^7^	76.4 (41.3, 130.7)	106.3 (66.2, 187.7)
Alpha-1-antitrypsin (mg/dl)^7^	13.2 (8.9, 21.9)	11.0 (8.9, 19.1)
**Fungal load**		
*Candida albicans* equivalent concentration (fg/µl)	26 (3, 131)	9 (3, 42)

1 Percentages.

2 Aspirin, excluded when prescribed in low-dose as anti-platelet treatment.

3 Seven current tobacco smokers, four with PD and three without.

4 Unified Parkinson’s Disease Rating Scale (normal, 0; severe 4 for each item).

5 Supporting Information.

6 Total of 90 participants in subgroup, 34 of which had PD.

7 Total of 67 participants in subgroup, 41 of which had PD.

### Faecal fungal load increased markedly from sixth decade of life

**Figure 2:**
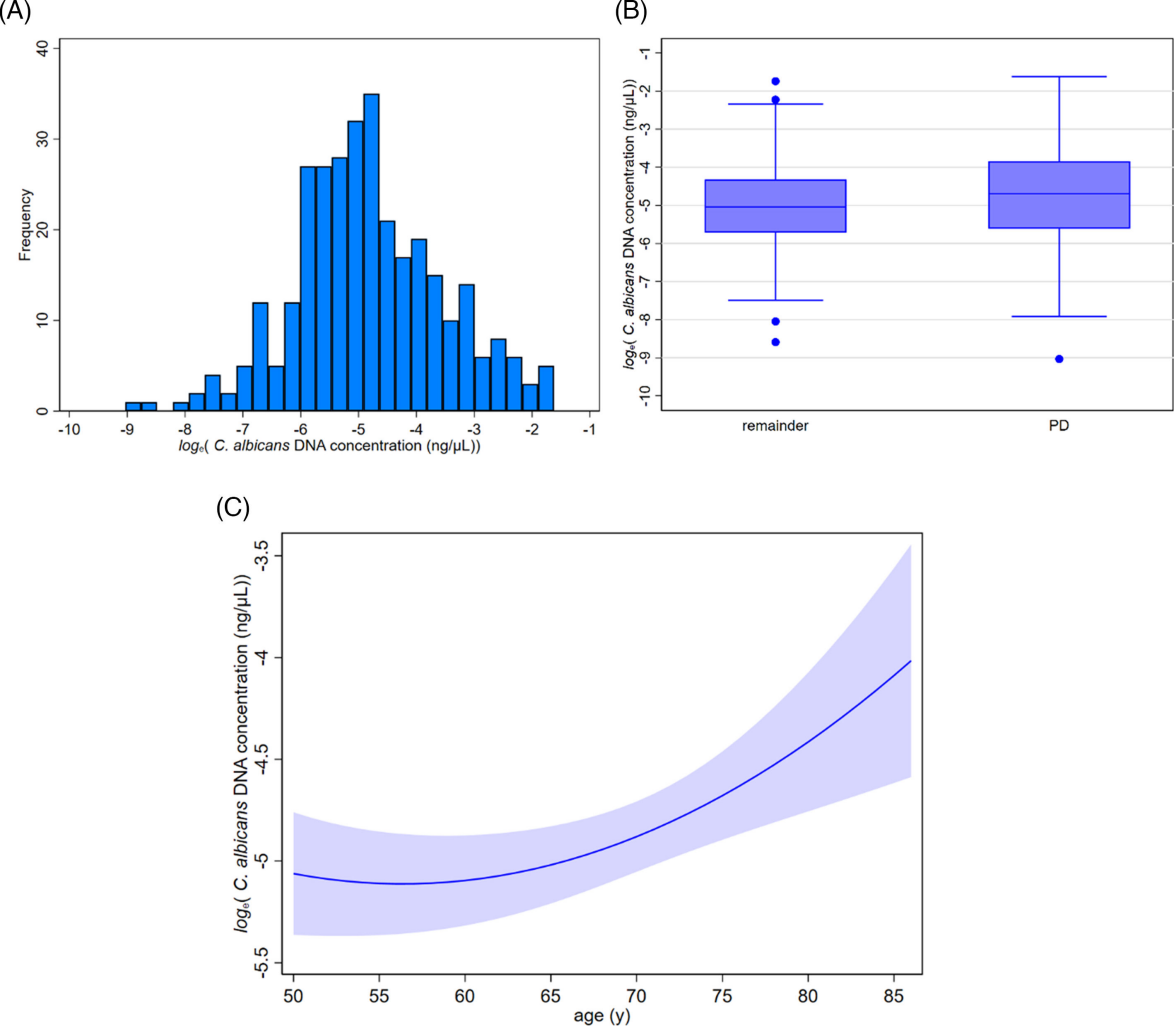
Fungal load. (**A**) Histogram of fungal load, expressed as *C. albicans* DNA equivalent concentration, against frequency, for all measurements. There were 108 measurements in 68 participants with PD, and 140 in 102 without. (**B**) Comparison of distribution of fungal load by PD-status. Box (median, upper and lower quartile) and whisker (1.5 times interquartile range) plots and outliers are shown. Median fungal load (5, 95 percentiles) was 20 (0.14, 6900) in diagnosed PD and 1.9 (0.18, 1800) fg/µl in the remainder. (**C**) Effect of age on fungal load. Regression line (95% CI) of age on fungal load expressed in terms of *C. albicans* equivalent concentration for entire group of 170 participants on a total of 248 occasions.

**Figure 2A** presents the fungal load data from all visits as a histogram. There was a total of 248 fungal load measurements. Sampling occasions (range 1–3 in PD, 1–4 in remainder) were a median of one year apart.

**Figure 2B** compares the distribution of fungal load in PD and the remainder. Significant differences by subgroup had been excluded in the remainder: 73 observations in 49 spouses/life partners of PD probands; 49 in 37 controls-proper, not having diagnosed PD nor sharing a house with someone with PD and without a blood-line family history of PD; and 18 in 16 controls with the family history of PD. During the short within-participant follow-up (mean 0.67 (minimum 0, maximum 3.32) years), there was no significant time trend in fungal load overall nor any difference by PD status or between remainder subgroups.

The age range of participants was from 50 to 86 years (**Table 1**). Fungal load increased with age (*P* = 0.13 for age and *P* = 0.002 for age^2^, with a combined effect of *P* = 0.004), irrespective of PD status with regard to either age term (**Figure 2C**). The turning point where fungal load started to increase was 56 years. The size of the ageing effect was large: an increase by around 60 (CI 16–119) % per decade after age 60 years. Gender had no significant effect on fungal load.

The number of antimicrobial courses in the last 3 years, and time since last course, had no effect on fungal load. Neither did concurrently a PPI or a NSAID. Time since cessation of tobacco smoking and total life-time experience of smoking were not associated with fungal load. History of cessation and duration was similar in PD and the remainder. Few participants currently smoked (**Table 1** ).

Both the presence of functional constipation and the number of colonic markers retained were doubled in PD, but the median stool consistency was normal irrespective of PD status (**Table 1**). Presence/absence of functional constipation, total number of transit markers retained in entire colon or its transverse segment and stool consistency rating (on specimen collection day) were unrelated to fungal load.

### Evidence faecal fungal load influenced by alkalinity of diet but not oral hygiene

Three components of nutritional intake (**Table 1** ), potential renal acid load (PRAL), vitamin β-carotene and inorganic constituent chloride were associated with fungal load (negative associations with PRAL and chloride, positive with β-carotene, *P* = 0.01, 0.03 and 0.04, respectively). In a multivariate model, adjusted for age and age^2^, only a weak contribution from PRAL remained, a –4.4 (CI –0.2, 8.8)% increment in fungal load for a 10-unit change in PRAL (*P* = 0.04).

The oral health impact profile (OHIP-14) was not associated with fungal load after age adjustments.

### Barrier dysfunction: inter-relationship of AAT and zonulin markers, but I-FABP independent

**Figure 3:**
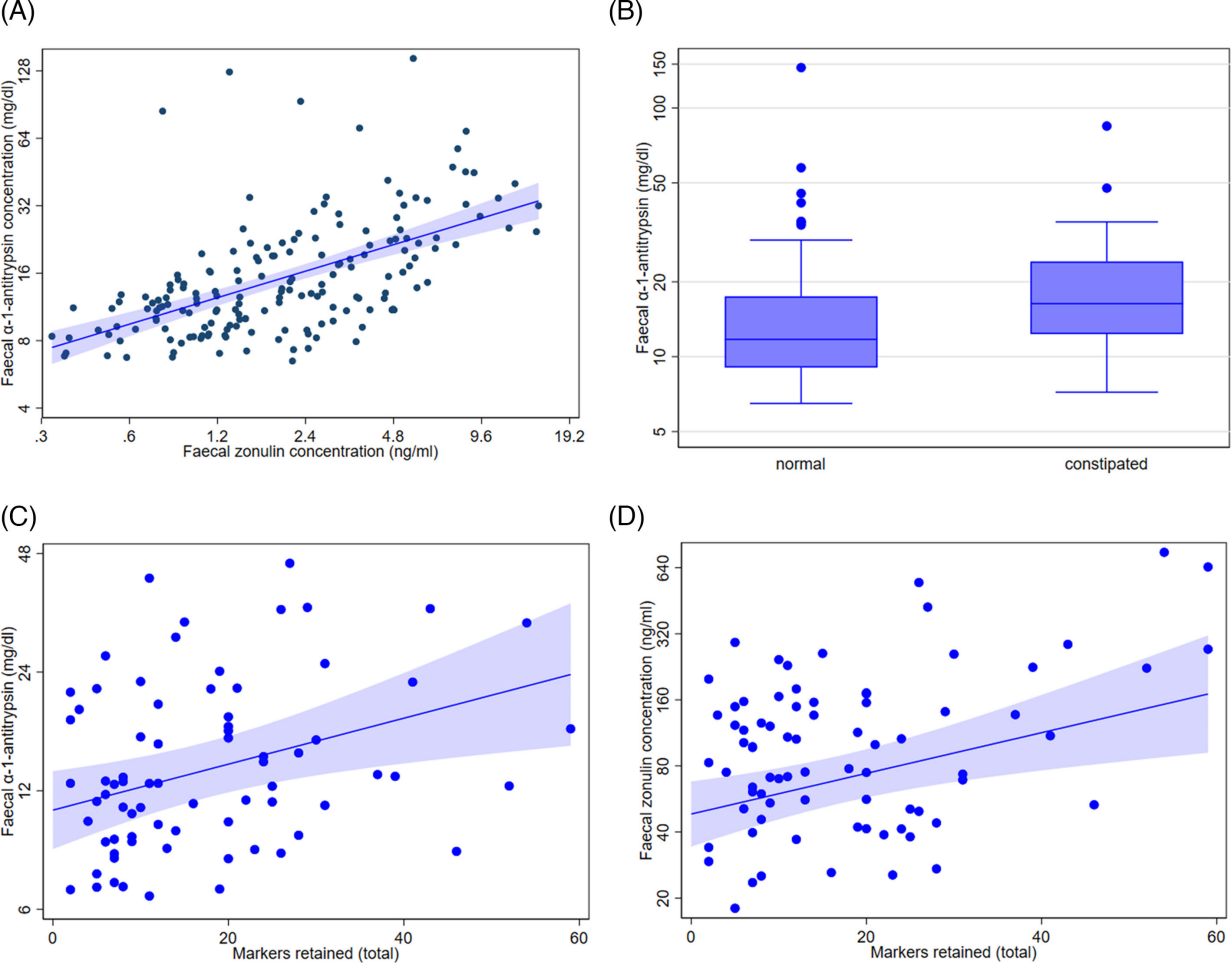
Barrier dysfunction markers. (**A**) Inter-relationship of two faecal markers of barrier dysfunction. Best fit regression line (95% CI) for α-1-antitrypsin (AAT) on zonulin (*n* = 176 occasions) was a linear relationship of log_e_ transformed concentrations. (For each AAT and zonulin concentration, the median [5, 95 percentiles] were 13.4 [8.0, 44.9] mg/dL and 1.55 [0.51, 5.77] ng/ml, respectively, in PD [84 occasions in 41]; 12.5 [7.0, 45.3] mg/dL and 2.17 [0.59, 8.50] ng/ml in remainder [92 occasions in 26].) (**B**) Distribution of α-1-antitrypsin (AAT) concentration in those with and without functional constipation. (**C**) AAT and (**D**) zonulin concentrations in relation to total number of colonic transit test markers retained.

There was a strong positive relationship (adjusted *r^2^* = 32%, *P*<0.001) between two faecal markers of barrier dysfunction, AAT and zonulin (**Figure 3A**). Notably, the serum concentration of barrier marker, I-FABP, was independent of that of faecal AAT and zonulin. (For I-FABP, the median [5, 95 percentiles] for 251 measurements was 434 [221, 915] pg/ml for PD [106 occasions in 34] and 414 [196, 848] pg/ml for the remainder [145 occasions in 56].)

There was no significant association of fungal load with the barrier dysfunction markers.

### Barrier dysfunction unaffected by PD status, but small intestinal marker increased with age

Intestinal barrier dysfunction markers were unaffected by PD status or age at diagnosis of PD. Age had a weak effect on one marker, serum I-FABP increasing by 6.3 (0, 13.0)% per decade, *P* = 0.05), with no interaction between PD status and age on concentration. Serum I-FABP concentration was higher in males (median [5, 95 percentiles] 451 [221, 1088] pg/ml) than in females (392 [195, 846] pg/ml) (Wilcoxon rank-sum test, *P* = 0.03), the gender effect being confined to PD (*P* = 0.01). Conversely, faecal zonulin was higher (*P* = 0.04) in females (2.13 [0.56, 11.0]) than in males (1.55 [0.50, 5.77] ng/ml), independent of PD status. AAT was unassociated with gender. Body mass index had no significant relationship to barrier dysfunction.

No within-participant temporal effects were detected, apart from a significant interaction between PD status and time (*P* = 0.04) for I-FABP, with a trend to increasing concentration in PD compared with the remainder (by mean 1.06 [0.98, 1.14] *cf* 0.95 [CI 0.89, 1.01] pg/ml/y). Mean time over which markers were monitored (average of two observations per marker) was 833 (289) days for I-FABP (50 participants), 510 (SD 236) days for AAT and zonulin (33).

### Marked increase in barrier dysfunction marker associated with proton pump inhibitors

In those taking a PPI, AAT was higher by mean (95% CI) by 151 (62, 287)% (*P* = 0.001, PD-status adjusted). More participants with PD (21%) than without (9%) were taking a PPI (Fisher’s exact test, *P* = 0.03).

For NSAIDs, more modest associations were seen with barrier markers: the serum I-FABP concentration was higher (by 27 [2, 58]%), but faecal AAT lower (by 63 [40, 98]%) (*P* = 0.04 and = 0.03, PD status adjusted). The proportion of those with PD (16%) and without (9%) taking NSAIDs was not significantly different.

History of antimicrobial treatment, time since cessation of tobacco smoking and total life-time experience of smoking were not associated with any of the markers.

### ‘Colonic’ barrier dysfunction markers associated with slow transit constipation, ‘small intestinal’ with looser stool

**Figure 3** illustrates associations between measures of constipation and the barrier dysfunction markers. **Figure 3B** shows that having functional constipation was more common, the higher the faecal AAT concentration (by 28 [CI 3, 51]% with a doubling in concentration (*P* = 0.025)). However, functional constipation was not significantly associated with zonulin. **Figure 3C and D** shows that the higher the AAT and the zonulin concentration, the more transit markers retained in the colon (*P* = 0.006 and 0.003, respectively). The problem appeared to be localised to the transverse colon, where 5 (2, 7) and 6 (2, 5)% of markers were retained with a doubling of concentrations (*P* = 0.001 and 0.007).

Neither the presence/absence of functional constipation nor the number of colonic transit markers retained was associated with serum I-FABP concentration. However, the stool consistency rating from 1 (hard lumps) to 7 (entirely liquid) [24] was higher (5 [1, 10]%) with a doubling in I-FABP concentration (*P* = 0.02).

### Faecal metabolome signature of small intestinal barrier dysfunction

**Figure 4:**
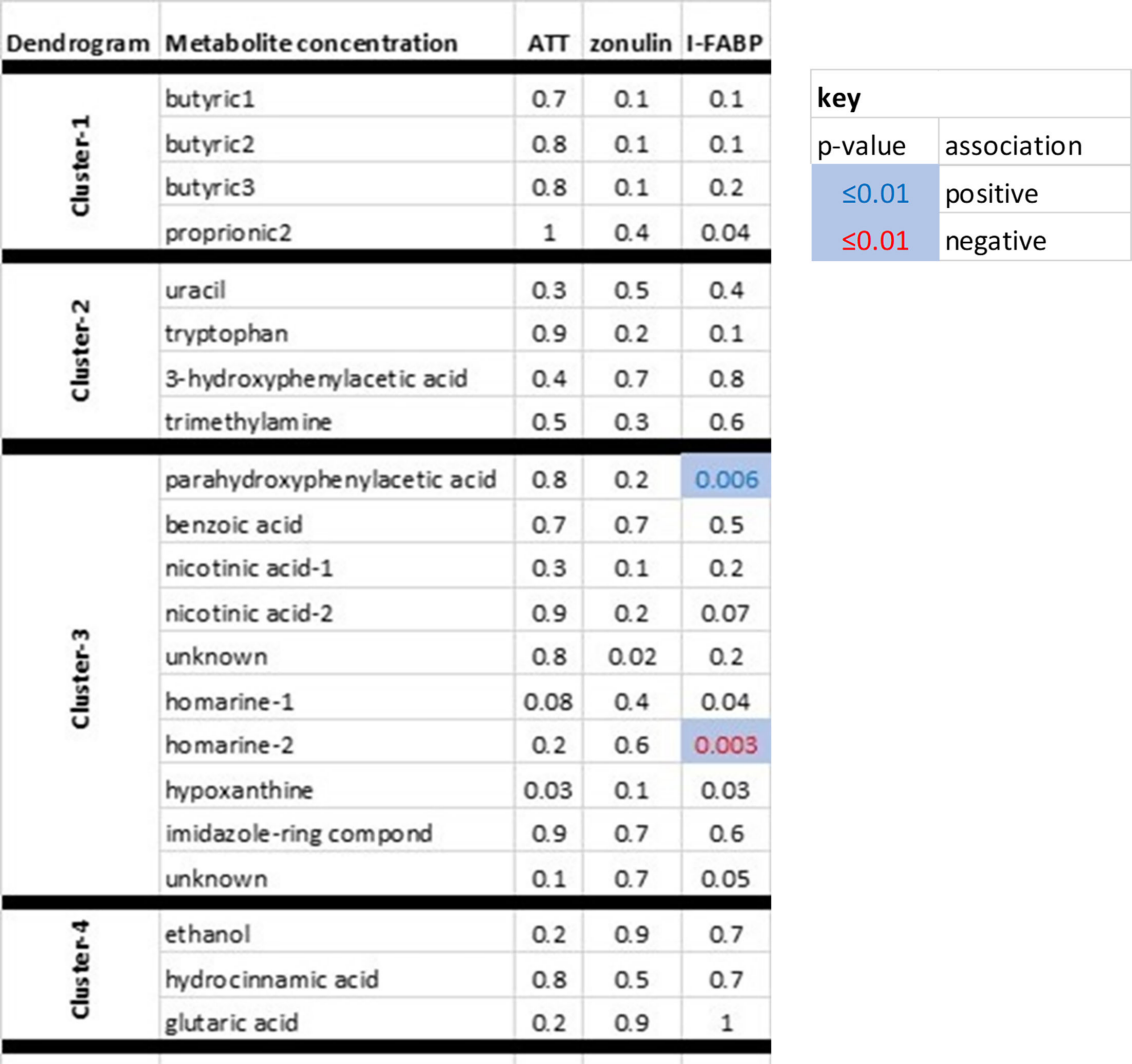
Faecal metabolite signature of barrier dysfunction. Heatmap of metabolite concentrations against those of intestinal markers, faecal α-1-antitrypsin (AAT) and zonulin, and serum intestinal fatty acid-binding protein (I-FABP), after correction for stool consistency and covariates. Analysis was irrespective of PD diagnostic threshold. Clustering refers to dendrogram of faecal metabolites discriminant for PD.^9^ (N.B.: Chemical shifts for butyric acid, nicotinic acid and homarine were complementary within cluster in discriminating.)

The association of faecal metabolites (previously demonstrated as discriminant for PD) [13] with the barrier disruption markers was confined to serum I-FABP, implicating the small intestine (**Figure 4**). At the 0.01 significance level, I-FABP concentration was positively associated with parahydroxyphenylacetic acid and negatively with one chemical shift in homarine. (The other shift in homarine was also negatively associated, but weakly so.)

There was no association of the metabolites with fungal load.

## Discussion

Does constipation matter? Looking at PD pathogenesis from the point of view of what comes first, constipation is an addressable contender. We associate colonic barrier dysfunction with both subjective (functional constipation) and objective (colonic transit) assessments of constipation. This provides a new focus for unravelling the pathogenesis. A compromised barrier may allow translocation of microbes, their components or products, which in turn drive a chronic systematic immune response. We postulate that superimposed age-related changes in the gut microbiome allow escalation down the PD pathway. Increases in faecal pathogenic fungi [4] together with decreases in useful faecal metabolites [13] may determine the pathophysiological trajectories in neglected constipation. Chronic electrophysiological features of slow transit constipation include a reduced number of intestinal cells of Cajal (‘pacemaker cells’ which mediate communication between autonomic nervous system and gastrointestinal smooth muscle) and reduced amounts of excitatory neurotransmitters within myenteric plexuses [25]. The first anatomical site to investigate with respect to PD would be the transverse colon [2]. Neurotransmitter modulation has been linked to the gut microbiota: bacteria have been shown to produce and/or consume neurotransmitters, including dopamine, noradrenaline, serotonin, gamma-aminobutyric acid, acetylcholine and histamine [26]. Indeed, changes in the gut microbiome could trigger the constipation forerunner of PD. At the other extreme, whether the severe constipation of later disease has a more profound effect on barrier function needs exploring.

We found no increase in the barrier dysfunction markers, faecal AAT or zonulin, with age in the older adults studied. However, a contrast of young (18–30 years) and older ( ≥70) groups brought out a significantly higher zonulin with age [27]. Remodelling of the colonic barrier with age has been described in the baboon [28]. We report an increase in I-FABP (highly expressed in small intestine [29]) with age in humans, noting that age-related remodelling of the small intestinal mucosa barrier has been described in the rat [30].

Is caeco-ileal reflux part of the chain of events? Two thirds of PD probands are, on presentation, lactulose hydrogen breath test positive for small-intestinal bacterial overgrowth (SIBO) [31]. A likely cause is caeco-ileal reflux from an overloaded right colon. Not only dysmotility but also retrograde peristaltic contractions are seen in slow transit constipation [25]. Overgrowth influences the circulating immunoinflammatory milieu, and biological gradients connect that milieu to PD phenotype [31].

The small intestine barrier dysfunction marker, I-FABP, was positively associated with the Bristol Stool Scale (the higher I-FABP, the looser the stool) and negatively with the concentration of an osmolyte, homarine, involved in maintaining cell integrity [32]. The strong positive association of I-FABP with the faecal metabolite p-hydroxyphenylacetic acid is compatible with higher concentrations of this metabolite being associated with SIBO [32], a cause of looser stool.

Irrespective of PD, small intestinal fungal overgrowth (SIFO) may co-exist with SIBO: a quarter of patients with unexplained gastrointestinal symptoms had SIFO, even in the non-immunocompromised [33]. The robust finding of a reduced blood lymphocyte count (by 24%) in PD probands, compared with controls, may, in part, represent translocation to the gut, rather than immunocompromise [34]. Given that the current tools and databases for analysing fungal species are underdeveloped relative to those for bacteria, the frequency of SIFO is likely to be underestimated.

Does the expansion of faecal fungal load underlie a systemic inflammatory response syndrome (SIRS) in PD? To our knowledge, this is the only report to date of an age-related increment in faecal fungal load in adult humans. The size of effect was large and progressive, around 60% increment per decade, and started in middle age. It is compatible with colonisation, but, since its magnitude did not influence the metabolite deficit, not with incremental encroachment on the bacteriome. Unlike the serum cytokine IL-6, the total fungal load did not show a premature ageing effect in PD. Discriminant pathogenic fungal taxa might.

Given the indication of migration of neutrophils to the intestinal mucosa in PD (see **Introduction**), it is important to explore microbial taxa associates of calprotectin. Given the low blood lymphocytes of PD [34], it is also important to study a marker of attraction of lymphocytes to mucosal surfaces, serum CCL20. This is particularly so in the light of the positive correlation between CCL20 and total colonic transit time [13], and the involvement of CCL20 and its receptor CCR6 in active inflammation in IBD [35].

The inverse relationship of fungal load to PRAL in diet, described, suggests that a higher intestinal pH is a promotor of fungal reproduction. However, the combined contribution of medicines (PPIs, histamine type-2 receptor antagonists, antibiotics), hypochlorhydria related to gastric atrophy and decreased bile acid synthesis may have a greater influence on intestinal pH than diet [36]. Moreover, faecal pH is higher in SIRS, a more pronounced effect being associated with bacteraemia [37]. This has been attributed, in part, to lower SCFA concentrations. A higher pH drives switching to fungal morphologies (e.g. hyphae) critical to disease progression, promoting virulence by facilitating penetration of host surfaces and hindering or evading immune responses [37]. Athough taking a cut-point of 40 years detected no difference in faecal pH in one study [38], change in pH may contribute to defining the turning point at 56 years in the age relationship to faecal fungal load. The substantial effect of PPI consumption, and hence gastrointestinal pH, found on barrier dysfunction requires further exploration.

The net effect of an increase in fungal load on intestinal inflammation needs to be considered in an inter-kingdom context. For example, positive correlations between *C. tropicalis*, *Escherichia* coli, and *Serratia marcescens* have been identified in Crohn’s disease (CD) patients and validated using *in vitro* biofilms [39]. In mice, susceptibility to colitis on eradication of commensal bacteria can be overturned by mono-colonisation with commensal enteric fungi [40], the fungi presumably appropriating the tonic microbial stimulation necessary to safeguard local and systemic immunity. However, invasive fungal infections are a severe complication of inflammatory bowel disease (IBD) treatment: most infections occurring within 12 months of starting treatment [41]. Understanding the 3.5 times greater odds of having microscopic colitis in PD than in the general population [42] may require exploration of the mycobiome.

Does the literature support manifestations of fungal infection being more common in PD or the mycobiome being different? It is apparent from our systematic review (Supplementary File S1) that more work on the alimentary tract mycobiome is needed in PD, with reference to age, diet, stool pH and constipation. The search combined two groups of keywords, one related to the disease target of PD, the other to the microbial target of fungi. Of the 556 articles screened, 33 were assessed for eligibility, but only 6 [43-48] met the inclusion criteria (**Figure 1**) of cross-sectional observational (cohort or case-control) study (with or without retrospective/prospective longitudinal data) and/or intervention study. They are classified according to biological sample type and anatomical location (Supplementary Table S1), results showing little evidence of fungal infection problems being more common in PD or of difference in mycobiota.

A faecal mycobiota dysbiosis is described in IBD with an increase in the phyla ratio, Basidiomycota/Ascomycota [49]. This was not seen in PD [43], where 86% of sequences mapped were Ascomycota, 12% Basidiomycota, the ratio being similar to that seen in healthy volunteers [50]. A reduced faecal fungal load in PD was claimed on the basis of fungal ITS2 DNA relative to bacterial 16S rRNA genes [43]. In a further faecal mycobiota study [44], three genera from the phylum Ascomycota, and one from Basidiomycota were more abundant in PD than in controls, whilst one from Ascomycota was less abundant. In neither study [43,44] was an overall age effect on fungal load reported. In saliva [45], there was increased abundance of three species from the phylum Ascomycota. The genus *Malassezia*, of phylum Basidiomycota, was more densely represented on skin in PD than in controls [46], irrespective of whether the skin was lesioned. This may relate to decreased facial skin motility and increased sebum excretion. The difference in fungal mycobiota was also addressed with respect to corpora amylacea, glycoprotein inclusions which accumulate in the brain during normal ageing and, to a greater extent, in some neurodegenerative diseases [47,48]. There were higher quantities of corpora amylacea in the samples from PD brains than from control brains, but evidence for these inclusions being a primary mycosis was weak.

We profile intestinal barrier dysfunction with respect to constipation, age and PD. Although a third of the variance in faecal AAT was explained by faecal zonulin, only 6% was explained by the intestinal inflammation marker, faecal calprotectin (personal communication AC, SMD, RJD). Serum I-FABP was independent of the other barrier dysfunction markers and of calprotectin.

In humans, I-FABP, a cytosolic protein that binds and transports fatty acids is highly expressed in the small intestine, particularly distally, expression being confined to mature enterocytes [29]. Its release into the circulation is a sensitive marker of small intestine enterocyte damage, intestinal ischaemia and necrotising enterocolitis. Serum I-FABP concentration is a useful clinical marker of CD, ileitis in ulcerative colitis (UC), mesenteric ischaemia and in early diagnosis of strangulated intestinal obstruction. In diarrhoea-predominant IBS patients, it correlates with increased small intestinal permeability as assessed by the lactulose/mannitol ratio in urine [51]. However, in PD, despite the relative frequency of SIBO [31], I-FABP concentration was not elevated.

Zonulin, a precursor to haptoglobin-2, is in the haptoglobin family of acute-phase reaction proteins [12,29]. Small intestinal exposure to bacteria is a trigger for zonulin release. It has been proposed to modulate intestinal permeability by disassembling the tight junctional protein complexes in the intestinal epithelium. Serum or plasma concentrations of zonulin have been suggested to mirror intestinal permeability in coeliac disease, type-1 diabetes, CD and even psychological distress. In PD [12], serum zonulin concentration has been reported as double that in controls. Faecal zonulin concentration has been reported as doubled in one PD study, but in another (as here), it was not significantly different from that in controls. Caution has been advised in interpreting results of some assays, due to lack of specificity for zonulin.

AAT is a circulating serine protease inhibitor [29]. Although primarily produced in the liver, it is also secreted by macrophages, enterocytes and Paneth cells. One of its main functions is to protect tissues from the proteolytic activity of immune cells, particularly neutrophils. Concentrations correlate with disease activity in CD. Faecal AAT clearance is a marker of clinical disease severity in IBD. In environmental enteric dysfunction (syndrome of inflammation, reduced absorbative capacity and impaired barrier function), AAT is a marker of intestinal permeability. Barrier disruption allows AAT to leak from serum into the intestine, where it is resistant to degradation by digestive enzymes. There is no normal faecal range against which to assess intestinal integrity, only one for diagnosing a protein-losing enteropathy. Systematic review [12] showed that higher faecal AAT concentrations have been reported in PD compared with controls, but this was not echoed here.

The gram-negative bacterial endotoxin, lipopolysaccharide (LPS), has been widely studied as a marker of translocation of microbes and their products [29,52]. It is secreted during normal outer membrane vesicle trafficking. It binds to the soluble acute phase protein, lipopolysaccharide-binding protein (LBP), presenting LPS to cell surface pattern receptors, CD14 and TLR4, involved in innate immunity. The most cited role of LPS is as a trigger for septic shock, but it is widely referenced in the pathogenesis of chronic diseases. In PD, immunohistochemistry of colonic biopsies showed direct evidence of bacterial translocation into the epithelium and lamina propria of the sigmoid colon [12]. Six studies showed lower circulating LBP in PD than in controls. Five of these also showed higher LPS in PD, the exception giving direct evidence of *E. coli* translocation into epithelium and lamina propria of the sigmoid colon. Cautions in interpretation are LPS heterogeneity, different trafficking pathways and differences in the host’s LPS inactivation mechanisms.

In conclusion, idiopathic Parkinsonism will encompass many sources of chronic peripheral inflammation, but, of these, one related to the almost unifying factor of constipation is a good starter. Constipation is associated with intestinal barrier dysfunction. Moreover, there is evidence that treating constipation can restore the microbiota [4] and, in PD, abate the temporal increase in rigidity [3]. The long-term effects of chronic constipation have not been widely studied, even though it affects 11–20% of the adult population [53]. It has been associated with symptoms ranging from psychological distress to impaired activity and reduced work productivity [54-56], but, of course, all these can be presentations of PD. Ensuring adequate fluid intake and dietary fibre supplementation are the first step treatment [57]. For those needing further treatment, regular osmotic laxatives are recommended, with stimulant laxatives when rescue is required. A shift to regarding constipation as ‘a chronic problem with serious consequences’ could have far-reaching consequences.

The accelerated increase in faecal fungal load with age demands, by its very magnitude, further understanding. At its core must be colonisation. Increased fungal content of swallowed saliva, change in diet with age, and a multifactorial influence on intestinal pH may contribute. The increased risk of colitis in PD [13], together with evidence that modulation of the mycobiota may be important in the development and severity of IBD [58], is reason to pursue. Could anti-fungal agents be of therapeutic use in managing barrier dysfunction? Is SIFO in PD more amenable to treatment than SIBO, and could that treatment have therapeutic benefit?

Here, we consider PD as peripheral-driven immunoinflammatory processes, mediated by the systemic, with scope for intervention, rather than focusing on the spread of aberrant protein deposition. Interventions are needed to unravel microbial associations, the aim being disease modification towards a more favourable trajectory. The phenotypic facets of PD do not present nor progress in parallel. Indeed, hierarchical cluster analysis of facets, measured irrespective of diagnostic divide, shows rigidity to be strikingly dissimilar from a cluster containing brady/hypokinesia, tremor and colonic transit time [13]. Predictors of the bimodal PD status may encompass different predictor sets for the facet clusters. The scope for facet separation is neatly illustrated by the randomised placebo-controlled trial of eradication of *Helicobacter pylori* in PD showing improvement in hypokinesia but deterioration in rigidity [59,60]: eradication, achieved by broad spectrum antimicrobial intervention, may have unlocked the next stage in the natural history.

### Limitations

We present a hypothesis on the aetiopathogenesis of PD. The supportive data are exploratory, laying foundations on which to build a description of transitions within the disease spectrum and longitudinal models for disease evolution. It sets the scene for greater depth in questioning, such as on the balance of pathogenic and commensal fungal taxa in the age-related escalation in load.

Restriction to faecal fungal load has allowed a clear-cut outcome, in terms of the large age effect, to unpick and understand. Mucosal biopsies may give a clearer picture than faeces as to the relevant fungal taxa. Interventional studies may build associations into cause/effect models. Since the facets of PD may have different drivers and mediators [61], their quantification may be a more useful outcome than PD status. An adaptive approach will allow expansion, in terms of cohorts and datasets of particular interest. Diet should be taken into account as a covariate in the future definition of the archetypical gut mycobiome of PD [18]. Differences in food colonisation may distinguish the mycobiome of vegetarians. Diseases and medication, which may predispose to fungal infection, are important considerations.

Here, faecal AAT and zonulin appear to describe colonic barrier dysfunction, serum I-FABP small intestinal, but there is no gold standard to measure intestinal barrier function. Therefore, it is important to combine different techniques to obtain a fit-for-purpose picture of the intestinal barrier [29].

## Supplementary material

Online supplementary material

## Data Availability

All supporting data are included within the main article and the supplementary file.

## Abbreviations

AAT: α-1-antitrypsin
I-FABP: intestinal fatty acid-binding protein
IL-6: interleukin-6
PD: Parkinson’s disease
PPIs: proton pump inhibitors
PRAL: potential renal acid load
SIBO: small-intestinal bacterial overgrowth
SIFO: small intestinal fungal overgrowth
SIRS: systemic inflammatory response syndrome
