# Supplementary material for: Interplay of constipation, intestinal barrier dysfunction and fungal exposome in aetiopathogenesis of Parkinson’s disease: hypothesis with supportive data
Source: Biochem J. 2025 Jun 11;482(12):807–21. doi: 10.1042/BCJ20240621 (PMC12235043; doi:10.1042/BCJ20240621)
Supplement: Online supplementary material [file bcj-482-12-BCJ20240621-supp1.docx]

**Supplementary file**

**Preliminary systematic review**

**Methods**

The review was conducted and reported according to the ‘Preferred Reporting Items for Systematic Reviews and Meta-Analyses (PRISMA)’ guidelines [1]. Ovid EMBASE, MEDLINE, Global Health and APA PsycInfo databases were searched, with restriction to full articles in or translated into English, published from 1974 in peer-reviewed scientific journals. The search combined two groups of keywords. One related to disease target (Parkinson’s disease; Parkinson with any alternate ending to word; idiopathic parkinsonism; parkinsonism), the other to the microbial target (fungi; fungal; mycobiome; fungal microbiome). A representative of each was required in title or abstract.

Figure 1 summarises the selection process. Included were cross-sectional observational (cohort or case-control) studies, with or without retrospective/prospective longitudinal data, and any intervention studies. Excluded were case reports, reviews, and meta-analyses. Reviewers (AP, CU) scanned the search results independently, and held consensus meetings to discuss discrepancies. Reviewer (RJD) was available to assess equivocal selections.

The information extracted from each article included: (1) citation; (2) type of study; (3) size and characteristics of cohorts; (4) definition of disease-status; (5) sampling site (nature and anatomical position) and methodology for fungal examination; (6) differences in fungal load or taxonomy between cohorts; (7) associations of fungal outcomes with disease duration and/or severity; (8) effect of relevant medication; and (9) geographical location of study.

Questions addressed were ‘How common is the (fungal infection) problem?’ and ‘What is the difference in mycobiota’ in PD [2]?


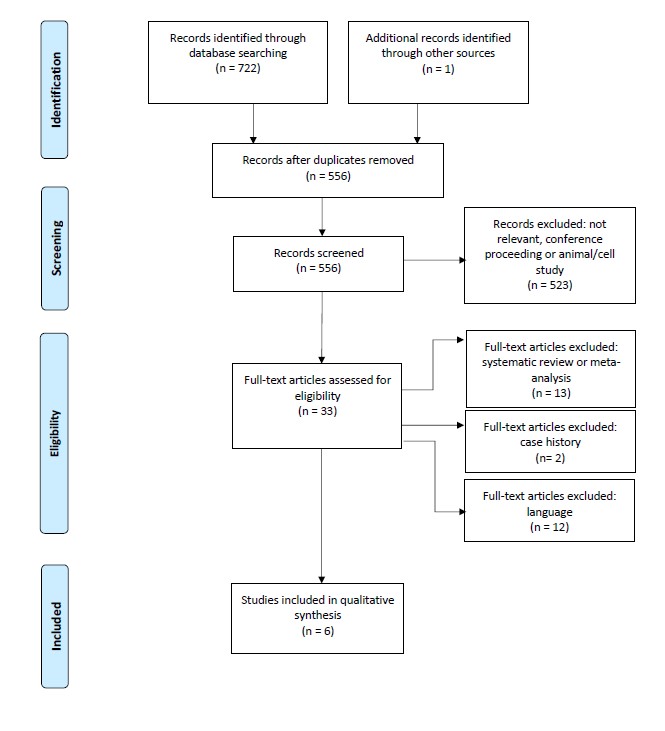


**Figure 1**  Article selection process in compliance with PRISMA guidelines.

**Results**

***Studies included and their design***

Of the 556 articles screened, 33 were assessed for eligibility, but only 6 [3-8] met inclusion criteria (3 from Europe, one from UK and 2 from North America) (Figure 1). They are classified according to sample type and anatomical location in Table 1. All but one (where antifungal immunostaining of post-mortem brain tissue not detailed in controls) [7] met Oxford Centre for Evidence-Based Medicine (OCEBM)^7^ evidence level 3. They were cross-sectional comparisons, with no interventions or longitudinal follow-up.

Regarding design, all those in PD-cohorts had a previous external diagnosis. Controls were usually defined by implication as not having diagnosed PD, with one study describing controls as age-matched [3]. Only one study [4] commented on previous or current antifungal and/or antibacterial treatments. No study addressed impact of gastrointestinal disease (e.g. irritable bowel syndrome) on findings, but constipation status was considered in two [3,4].

***Is fungal infection problem more common in PD***

Possible clinical manifestations of fungal infection are addressed in only one study: the suspected pathogen was more abundant in PD but not the skin lesions [6]. It was first questioned whether the presence/absence and severity of seborrheic dermatitis are associated with fungal infection. Seborrheic dermatitis was more common in males, and with increasing age, irrespective of PD-status. Surprisingly, it was in those without PD that seborrheic dermatitis was more severe in terms of percentage of skin area affected, although not in severity. However, a higher density of *Malassezia* was found in PD, irrespective of whether the skin was lesioned. This may relate to decreased facial skin motility and increased sebum excretion. Seven species of *Malassezia* were cultured from forehead skin and identified by microscopic characteristics, presence of a yeast enzyme and ability to utilize different substrates. The most abundant, in both seborrheic dermatitis and PD were *M. globosa*, *M. furfur* and *M. obtuse.*

***What is the difference in fungal mycobiota in PD***

The ratio of fungal to bacterial DNA was reduced in PD, even after exclusion of those with low fungal genomic content (<100 reads) in one of the two studies of faecal mycobiome [3]: here, a ‘denominator effect’ artefact, due to small intestinal bacterial overgrowth being more common in PD, was proposed. No fungal taxonomic differences by PD-status were found. In particular, similar faecal abundance of *Malassezia* (see above [6]) was noted in the two cohorts. Dietary assessment was confined to an annual food frequency questionnaire. In the other study [4], *Saccharomyces* was less abundant in PD, four other genera (Table 1) being more abundant. The influence of diet was not described. Alpha-diversity was greater in PD, irrespective of constipation or PD severity. However, primary controls were from a bowel cancer screening programme (with normal colonoscopy). In neither study was an overall age effect on fungal load reported [3,4].

The only study examining saliva showed an increase in abundance of three fungal species in PD compared with controls (*Candida albicans*, *C. dubliniensis* and *Saccharomyces cerevisiae*), but no significant differences in α- or β-diversity by PD-status [5]. Active dental caries, periodontal disease, and diseases of the naso- and oro-pharyngeal cavities were exclusions.

Corpora amylacea are glycoprotein inclusions which accumulate in brain during normal ageing and, to a greater extent, in some neurodegenerative diseases, but evidence for these inclusions being a primary pre-mortem mycosis is weak [5,6]. There were higher quantities of corpora amylacea in the samples from PD brains, than from control brains [7]. Immunostaining for a rabbit polyclonal antibody, against three *Candida* species (*C. famata*, *C. albicans*, *C. glabrata*), *Neocamarosporium betae* and *Syncephalastrum racemosum* was illustrated in all 6 PD brains. (*N. betae* is a ubiquitous mould, found in decaying plant material, with airborne spores. *S. racemosum* has been associated with nail disease, which appears common in PD.) No information on time to post-mortem or of storage was given. In a second study by the same group [8], it was specified that frozen tissue was handled with sterile instruments in a laminar flow hood. Direct visualization for fungal morphology indicated that fungal presence was greater in PD than in controls. Immunostaining for chitin, a component of fungal cell wall, showed intracellular and intranuclear staining in PD brain, but not in control brain. Most of the fungal species identified were of the genera *Botrytis*, *Candida*, *Fusarium* and *Malassezia*, spatial distribution between brain regions being different in PD from that in controls.

**Table 1 Studies meeting inclusion criteria**

| **Authors** | **Number of participants** | **Microbial detection method**  (sample delivery and storage) | **Findings** |
| --- | --- | --- | --- |
| **Faecal** | | | |
| Cirstea et al., 2020 [3] | 64 PD;  42 controls  with detectable fungal genomic content. | ITS2 qPCR for fungal load and sequencing.  16S rRNA amplicon sequencing.  (samples mailed in stabilisation buffer, stored at -80°C) | Reduced fungal load relative to bacterial DNA in PD. No fungal taxonomic differences between PD and controls. |
| De Pablo-Fernandez et al., 2022 [4] | 24 PD;  20 ‘primary’ controls | 18S rRNA gene amplicons sequenced.  (samples delivered within 12 h, or frozen at home for 24h, stored at -80°C) | Four genera more abundant in PD, one less, α-diversity being greater in PD. |
| **Salivary** | | | |
| Mihaila et al., 2019 [5] | 48 early-stage PD;  36 controls | High-throughput RNA sequencing (NextSeq 500)  (saliva collection on site 30 minutes after last food or drink consumption.) | Increased abundance of *Candida albicans*, *C. dubliniensis* and *Saccharomyces cerevisiae* in PD, but no difference in α- or β-diversity. |
| **Skin of forehead** | | | |
| Arsic Arsenijevic et al., 2014 [6] | 30 PD;  60 controls with seborrheic dermatitis;  60 controls without seborrheic dermatitis | Culture with density expressed as number of colony forming units per tape (skin sample from forehead using stripping tape.) | Highest density of *Malassezia* in lesioned skin in PD, irrespective of whether skin lesioned. *M. globose* was most abundant species in PD. |
| **Post-mortem brain tissue** | | | |
| Pisa et al., 2016 [7] | 6 PD  5 controls | Immunostaining of brain with anti-fungal antibodies.  (post-mortem brain tissue sections from 6 different regions.) | Immunostaining-positivity of ‘corpora amylacea’ inclusions in all PD brains using a rabbit polyclonal anti-fungal antibody. Inclusions less abundant in controls and immunoreactivity modest. |
| Pisa et al., 2020 [8] | 6 PD  4 controls | As above, plus (i) nested PCR assay (ITS-1 and ITS-2 regions), (ii) next-generation sequencing, and (iii) 10 different brain regions plus spinal cord sampled. | Positive immunostaining for fungal morphology in PD, but not in controls. Spatial distribution of fungal species different in PD from controls. |

**References**

1. Page M J, McKenzie JE, Bossuyt PM, Boutron I, Hoffmann TC, Mulrow CD et al. The PRISMA 2020 statement: an updated guideline for reporting systematic reviews *BMJ*2021;372:n71
2. Howick J, Chalmers I, Glasziou P, et al. OCEBM levels of evidence working group. *The Oxford 2011 Levels of Evidence*. Oxford Centre for Evidence-Based Medicine.
3. Cirstea MS, Sundvick K, Golz E, et al. The gut mycobiome in Parkinson’s disease. *J Parkinson's Disease.* 2020:11;153-158.
4. De Pablo-Fernandez E, Gebeyehu GG, Flain L, et al. The faecal metabolome and mycobiome in Parkinson's disease. *Parkinsonism Relat Disord*. 2022;95:65-69.
5. Mihaila D, Donegan J, Barns S, et al. The oral microbiome of early stage Parkinson’s disease and its relationship with functional measures of motor and non-motor function. *PLoS One.* 2019;14:e0218252.
6. Arsic Arsenijevic VS, Milobratovic D, Barac AM et al*.* A laboratory-based study on patients with Parkinson’s disease and seborrheic dermatitis: the presence and density of *Malassezia* yeasts, their different species and enzymes production. *BMC Dermatol.* 2014;14:5.
7. Pisa D, Alonso R, Carrasco L. Parkinson's disease: a comprehensive analysis of fungi and bacteria in brain tissue. *International J Biological Sciences.* 2020;16:1135.
8. Pisa D, Alonso, R, Rábano A, Carrasco L. Corpora amylacea of brain tissue from neurodegenerative diseases are stained with specific antifungal antibodies. *Frontiers Neuroscience.* 2016;10:86.

**Data tables**

**Table 1** Faecal fungal load from all visits

| Unique individuals in numerical order* | occasion | age (years) | sex M=male F=female | fungal load  *C. albicans* equivalent (fg/µl) |
| --- | --- | --- | --- | --- |
| 1 | 1 | 53 | F | 143.4 |
| 1 | 2 | 55 | F | 1.7 |
| 1 | 3 | 57 | F | 0.3 |
| 2 | 2 | 55 | M | 5936.3 |
| 3 | 1 | 54 | F | 39.9 |
| 4 | 1 | 55 | M | 2.8 |
| 4 | 2 | 58 | M | 727.4 |
| 5 | 2 | 67 | F | 681.4 |
| 6 | 1 | 82 | M | 24.8 |
| 6 | 2 | 84 | M | 14.4 |
| 6 | 3 | 85 | M | 18.5 |
| 7 | 1 | 78 | F | 136.8 |
| 7 | 2 | 80 | F | 30.4 |
| 7 | 3 | 81 | F | 8726.4 |
| 8 | 2 | 69 | M | 3022.8 |
| 8 | 3 | 71 | M | 403.8 |
| 9 | 2 | 61 | F | 28.8 |
| 10 | 2 | 70 | M | 4.0 |
| 10 | 3 | 72 | M | 1015.0 |
| 11 | 2 | 74 | F | 14.8 |
| 11 | 3 | 75 | F | 12.8 |
| 12 | 1 | 68 | M | 142.6 |
| 12 | 2 | 71 | M | 1.3 |
| 13 | 1 | 72 | M | 113.7 |
| 13 | 2 | 75 | M | 45.9 |
| 14 | 1 | 70 | F | 8.1 |
| 14 | 2 | 72 | F | 117.8 |
| 15 | 1 | 73 | M | 159.1 |
| 15 | 2 | 75 | M | 471.0 |
| 15 | 3 | 76 | M | 124.0 |
| 16 | 1 | 66 | F | 130.5 |
| 17 | 1 | 64 | M | 847.9 |
| 18 | 1 | 52 | F | 1.3 |
| 19 | 1 | 65 | M | 3.0 |
| 19 | 2 | 67 | M | 20.1 |
| 20 | 1 | 65 | F | 1272.8 |
| 20 | 2 | 68 | F | 1181.9 |
| 21 | 2 | 74 | M | 2732.8 |
| 22 | 1 | 72 | F | 14.9 |
| 22 | 2 | 74 | F | 3888.9 |
| 23 | 2 | 72 | M | 83.4 |
| 23 | 3 | 73 | M | 2.3 |
| 24 | 2 | 56 | F | 4.7 |
| 25 | 2 | 78 | M | 23971.6 |
| 26 | 1 | 73 | F | 1.1 |
| 26 | 2 | 75 | F | 6852.9 |
| 26 | 3 | 76 | F | 16.4 |
| 27 | 1 | 62 | M | 4.7 |
| 27 | 1 | 62 | M | 7.9 |
| 28 | 1 | 61 | F | 1.0 |
| 28 | 2 | 64 | F | 12.6 |
| 29 | 1 | 69 | M | 161.8 |
| 29 | 2 | 71 | M | 195.1 |
| 29 | 3 | 72 | M | 514.3 |
| 30 | 2 | 69 | F | 4.6 |
| 30 | 3 | 70 | F | 43.3 |
| 31 | 3 | 64 | M | 0.1 |
| 31 | 4 | 65 | M | 22.7 |
| 32 | 1 | 61 | F | 32.6 |
| 32 | 2 | 62 | F | 2.4 |
| 32 | 3 | 63 | F | 1.9 |
| 33 | 1 | 58 | F | 10.5 |
| 34 | 1 | 75 | M | 12.2 |
| 34 | 2 | 77 | M | 3635.1 |
| 34 | 3 | 78 | M | 3.4 |
| 35 | 2 | 73 | F | 342.4 |
| 35 | 3 | 74 | F | 0.2 |
| 36 | 1 | 74 | M | 21.7 |
| 36 | 2 | 76 | M | 19.5 |
| 36 | 3 | 77 | M | 0.1 |
| 37 | 1 | 77 | F | 319.5 |
| 37 | 2 | 79 | F | 0.1 |
| 37 | 3 | 80 | F | 5.5 |
| 38 | 1 | 73 | F | 53.1 |
| 38 | 2 | 75 | F | 3.8 |
| 38 | 3 | 76 | F | 895.0 |
| 39 | 2 | 82 | M | 0.1 |
| 39 | 3 | 84 | M | 0.7 |
| 39 | 4 | 84 | M | 521.6 |
| 40 | 1 | 73 | M | 21.8 |
| 41 | 1 | 64 | F | 17.5 |
| 42 | 1 | 66 | M | 0.6 |
| 43 | 1 | 67 | F | 1.4 |
| 43 | 2 | 68 | F | 1.1 |
| 44 | 2 | 77 | M | 2639.0 |
| 45 | 1 | 69 | F | 3.6 |
| 45 | 2 | 71 | F | 15.8 |
| 45 | 4 | 73 | F | 1.1 |
| 46 | 1 | 65 | M | 0.0 |
| 46 | 2 | 67 | M | 0.0 |
| 46 | 3 | 68 | M | 2.8 |
| 46 | 4 | 68 | M | 80.7 |
| 47 | 1 | 65 | F | 14.6 |
| 48 | 1 | 62 | M | 2.4 |
| 48 | 2 | 63 | M | 4.6 |
| 48 | 3 | 65 | M | 0.0 |
| 49 | 1 | 78 | M | 131.2 |
| 50 | 1 | 66 | F | 3.1 |
| 51 | 1 | 72 | M | 1443.7 |
| 51 | 2 | 76 | M | 257.1 |
| 52 | 1 | 72 | F | 988.7 |
| 52 | 2 | 75 | F | 16.0 |
| 53 | 1 | 59 | F | 1173.7 |
| 53 | 3 | 61 | F | 6.7 |
| 53 | 4 | 62 | F | 403.0 |
| 54 | 1 | 61 | F | 596.9 |
| 54 | 2 | 63 | F | 10.5 |
| 55 | 1 | 62 | M | 28.1 |
| 56 | 1 | 68 | F | 55.9 |
| 57 | 1 | 79 | F | 1009.3 |
| 58 | 2 | 83 | M | 721.9 |
| 59 | 3 | 69 | M | 1752.5 |
| 60 | 1 | 63 | F | 9.1 |
| 60 | 2 | 65 | F | 19.5 |
| 60 | 3 | 66 | F | 3679.9 |
| 61 | 1 | 58 | M | 28.6 |
| 61 | 3 | 60 | M | 224.6 |
| 62 | 1 | 67 | M | 0.8 |
| 62 | 2 | 68 | M | 1.7 |
| 62 | 3 | 69 | M | 0.1 |
| 63 | 1 | 59 | F | 0.1 |
| 64 | 1 | 67 | M | 3.2 |
| 65 | 1 | 85 | M | 1.2 |
| 65 | 3 | 86 | M | 0.6 |
| 66 | 1 | 81 | F | 783.6 |
| 67 | 1 | 61 | M | 35.4 |
| 68 | 1 | 57 | F | 3.4 |
| 69 | 1 | 50 | M | 12.1 |
| 69 | 3 | 53 | M | 59.5 |
| 70 | 1 | 68 | F | 21.8 |
| 70 | 2 | 69 | F | 0.0 |
| 71 | 1 | 69 | M | 7.0 |
| 72 | 1 | 76 | F | 0.4 |
| 73 | 1 | 75 | F | 5.0 |
| 74 | 1 | 69 | F | 124.4 |
| 75 | 1 | 72 | F | 2.2 |
| 76 | 1 | 57 | F | 49.2 |
| 76 | 2 | 58 | F | 31.8 |
| 77 | 1 | 57 | M | 6.4 |
| 77 | 2 | 58 | M | 246.5 |
| 77 | 3 | 59 | M | 6.5 |
| 78 | 1 | 74 | M | 0.0 |
| 79 | 1 | 72 | F | 20.1 |
| 79 | 2 | 73 | F | 33.0 |
| 80 | 2 | 62 | M | 74.7 |
| 81 | 1 | 64 | F | 0.1 |
| 82 | 1 | 75 | M | 56.4 |
| 83 | 1 | 50 | F | 43.0 |
| 84 | 1 | 50 | M | 9.3 |
| 84 | 2 | 51 | M | 18.4 |
| 85 | 1 | 74 | M | 238.0 |
| 86 | 1 | 67 | F | 18168.7 |
| 86 | 2 | 69 | F | 19.4 |
| 87 | 1 | 73 | M | 225.5 |
| 88 | 1 | 73 | F | 0.3 |
| 89 | 1 | 65 | M | 25.6 |
| 90 | 1 | 68 | M | 0.0 |
| 91 | 1 | 68 | F | 6.4 |
| 92 | 2 | 77 | F | 18.6 |
| 93 | 1 | 58 | F | 3.4 |
| 94 | 2 | 67 | F | 1.0 |
| 95 | 1 | 60 | M | 1.3 |
| 96 | 1 | 83 | M | 8.6 |
| 97 | 1 | 63 | F | 14.0 |
| 97 | 2 | 64 | F | 4.0 |
| 98 | 1 | 70 | M | 0.0 |
| 99 | 1 | 70 | F | 11.7 |
| 100 | 1 | 67 | F | 5.8 |
| 101 | 1 | 71 | M | 77.2 |
| 102 | 1 | 61 | F | 1.8 |
| 103 | 1 | 61 | M | 31.7 |
| 104 | 1 | 71 | M | 0.2 |
| 105 | 1 | 74 | F | 14229.3 |
| 106 | 1 | 75 | M | 17.1 |
| 107 | 1 | 60 | M | 0.9 |
| 108 | 1 | 58 | F | 0.3 |
| 109 | 1 | 74 | M | 3.2 |
| 109 | 2 | 75 | M | 33.9 |
| 110 | 1 | 73 | F | 6.3 |
| 111 | 1 | 66 | M | 47.6 |
| 112 | 1 | 66 | F | 100.4 |
| 112 | 2 | 67 | F | 1.9 |
| 113 | 1 | 69 | M | 108.7 |
| 113 | 2 | 70 | M | 169.0 |
| 114 | 1 | 78 | M | 1.6 |
| 115 | 1 | 67 | M | 3.9 |
| 115 | 2 | 68 | M | 2.0 |
| 116 | 1 | 68 | F | 8.4 |
| 117 | 1 | 51 | M | 12.6 |
| 117 | 2 | 52 | M | 170.3 |
| 118 | 1 | 70 | F | 2.6 |
| 119 | 1 | 66 | F | 17.4 |
| 120 | 1 | 69 | M | 7.2 |
| 121 | 1 | 72 | M | 43.8 |
| 122 | 1 | 71 | F | 3.8 |
| 122 | 2 | 72 | F | 18.9 |
| 123 | 1 | 66 | F | 2.5 |
| 124 | 1 | 75 | M | 442.9 |
| 125 | 1 | 69 | F | 3.5 |
| 126 | 1 | 60 | M | 4.8 |
| 127 | 1 | 72 | M | 1.9 |
| 128 | 1 | 71 | F | 0.8 |
| 129 | 1 | 67 | M | 997.7 |
| 130 | 1 | 67 | F | 2.9 |
| 131 | 1 | 72 | M | 9.1 |
| 132 | 1 | 68 | F | 2.3 |
| 133 | 1 | 77 | M | 185.8 |
| 134 | 1 | 72 | F | 4.2 |
| 135 | 1 | 70 | M | 47.4 |
| 136 | 1 | 72 | M | 37.4 |
| 137 | 1 | 66 | F | 42.2 |
| 138 | 1 | 52 | M | 11.4 |
| 139 | 1 | 53 | M | 11.7 |
| 140 | 1 | 56 | M | 0.2 |
| 141 | 1 | 64 | M | 9.9 |
| 142 | 1 | 63 | M | 6.9 |
| 143 | 1 | 62 | F | 9.1 |
| 144 | 1 | 69 | F | 69.8 |
| 145 | 1 | 70 | M | 0.5 |
| 146 | 1 | 77 | M | 415.0 |
| 147 | 1 | 65 | F | 1.3 |
| 148 | 1 | 78 | F | 99.4 |
| 149 | 1 | 66 | M | 2.5 |
| 150 | 1 | 66 | F | 0.0 |
| 151 | 1 | 69 | M | 22.4 |
| 152 | 1 | 66 | M | 9.0 |
| 153 | 1 | 74 | M | 230.9 |
| 154 | 1 | 50 | F | 22.5 |
| 155 | 1 | 52 | M | 0.3 |
| 156 | 1 | 59 | F | 9.9 |
| 157 | 1 | 71 | F | 0.3 |
| 158 | 1 | 65 | M | 83.4 |
| 159 | 1 | 59 | F | 7.1 |
| 160 | 1 | 57 | F | 11.5 |

* repeats in same individual not included

**Table 2** Intestinal barrier markers measurements

| Unique individuals in numerical order* | Faecal alpha-1-antitrypsin (mg/dl) | Serum intestinal fatty acid-binding protein  (pg/ml) | Faecal zonulin  (ng/ml) |
| --- | --- | --- | --- |
| 1 | 12 | 235 | 98 |
| 2 |  | 205 |  |
| 3 | 10 | 196 | 71 |
| 4 | 8 | 321 | 53 |
| 5 |  | 433 |  |
| 6 | 16 |  | 56 |
| 7 |  | 173 |  |
| 8 | 19 | 206 | 136 |
| 9 |  | 915 |  |
| 10 |  | 327 |  |
| 11 |  | 436 |  |
| 12 | 18 | 789 | 83 |
| 13 | 9 | 417 | 56 |
| 14 | 23 | 532 | 110 |
| 15 | 20 |  | 106 |
| 16 |  | 420 | 645 |
| 17 | 16 | 392 | 70 |
| 18 | 22 | 480 | 292 |
| 19 |  | 845 |  |
| 20 | 11 | 200 | 46 |
| 21 | 13 | 470 | 137 |
| 22 | 22 | 579 | 78 |
| 23 |  | 922 |  |
| 24 |  | 878 |  |
| 25 |  | 496 |  |
| 26 |  | 323 |  |
| 27 | 12 | 468 | 51 |
| 28 | 16 | 478 | 258 |
| 29 |  | 846 |  |
| 30 | 21 |  | 199 |
| 31 |  | 454 |  |
| 32 |  | 373 |  |
| 33 |  | 382 |  |
| 34 |  | 800* |  |
| 35 |  | 198 |  |
| 36 |  | 551 |  |
| 37 |  | 271 |  |
| 38 | 42 | 730 | 229 |
| 39 | 17 | 418 | 272 |
| 40 |  | 355 |  |
| 41 | 12 | 562 | 51 |
| 42 | 15 | 191 | 44 |
| 43 | 24 | 359 | 114 |
| 44 |  | 825 |  |
| 45 |  | 351 |  |
| 46 | 8 | 306 | 50 |
| 47 | 8 | 460 | 41 |
| 48 | 32 | 289 | 260 |
| 49 |  | 783 |  |
| 50 |  | 729 |  |
| 51 | 7 | 384 | 42 |
| 52 | 7 | 359 | 29 |
| 53 | 11 | 634 | 39 |
| 54 | 13 | 392 | 125 |
| 55 | 11 |  | 38 |
| 56 | 12 | 338 | 223 |
| 57 | 6 | 584 | 108 |
| 58 | 7 | 925 | 123 |
| 59 | 7 | 848 | 61 |
| 60 | 35 |  | 286 |
| 61 |  | 823 | 75 |
| 62 | 17 | 169 | 155 |
| 63 | 13 | 464 | 60 |
| 64 | 13 |  | 34 |
| 65 |  | 562 |  |
| 66 | 16 | 511 | 149 |
| 67 | 26 | 257 | 157 |
| 68 | 9 |  | 40 |
| 69 | 32 | 390 | 752 |
| 70 | 14 | 800* | 41 |
| 71 | 10 | 224 | 75 |
| 72 | 35 | 710 | 548 |
| 73 | 8 | 529 | 97 |
| 74 | 9 | 695 | 102 |
| 75 | 35 | 486 | 141 |
| 76 | 13 | 390 | 179 |
| 77 | 10 | 582 | 37 |
| 78 | 9 | 448 | 25 |
| 79 | 45 | 210 | 423 |
| 80 | 9 | 800* | 121 |
| 81 | 11 | 876 | 166 |
| 82 | 18 | 268 | 170 |
| 83 | 15 | 899 | 106 |
| 84 | 23 | 410 | 244 |
| 85 | 11 | 483 | 149 |
| 86 | 9 | 396 | 136 |
| 87 | 29 | 605 | 156 |
| 88 | 9 | 568 | 27 |
| 89 | 13 | 396 | 225 |
| 90 | 7 | 520 | 25 |
| 91 | 25 | 557 | 73 |
| 92 | 13 | 864 | 72 |
| 93 | 9 | 241 | 54 |
| 94 | 11 | 800* | 26 |
| 95 | 7 | 427 | 18 |
| 96 | 10 | 512 | 75 |
| 97 | 13 | 414 | 116 |
| 98 | 8 | 342 | 64 |
| 99 | 17 | 292 | 172 |
| 100 |  | 312 | 24 |
| 101 | 22 | 417 | 100 |
| 102 | 11 | 754 | 69 |

* Right censored

**Definitions**

Definitions of previously reported [1] data, integrated into the analysis:-

1. **Nutritional intake**. Nutritional intake had been measured using a standard consecutive 5-day unweighted food, drink and supplement diary, and entered into an online nutritional analysis software (Nutritics, Research Edition, v5.021; Dublin).
2. **Colonic transit time**. Colonic transit had been assessed using Transit-Pellets (Medifactia, Sweden). Participants were requested to swallow a pellet containing ten ring-shaped radio-opaque barium markers, at 10:00 h. on five consecutive days. On day 6, a pellet containing five rod-shaped markers was swallowed at 10:00 h and another at 22.00 h (for assessment of rapid transit). An abdominal radiograph was taken on day 7, approximately 12 hours after the last pellet. Retained markers were counted for each ascending, transverse, descending, and sigmoid colon and for rectum by same specialist consultant gastroenterological radiologist.
3. **Faecal metabolome concentrations**. Faecal aqueous extracts had been obtained from one sample tube per person immediately on delivery and stored in aliquots of ≥0.5 ml at –80°C for the NMR spectral metabolomic analysis.
4. Augustin A, Guennec AL, Umamahesan C, et al. Faecal metabolite deficit, gut inflammation and diet in Parkinson's disease: Integrative analysis indicates inflammatory response syndrome. *Clin Transl Med*. 2023;13:e1152.
